# Cortical plasticity in episodic and chronic cluster headache

**DOI:** 10.1016/j.nicl.2014.10.003

**Published:** 2014-10-18

**Authors:** Steffen Naegel, Dagny Holle, Nathalie Desmarattes, Nina Theysohn, Hans-Christoph Diener, Zaza Katsarava, Mark Obermann

**Affiliations:** a Department of Neurology, University of Duisburg Essen, Hufelandstr. 55, Essen 45122, Germany; b Department of Diagnostic and Interventional Radiology and Neuroradiology, University Hospital Essen, Hufelandstr. 55, Essen 45122, Germany

**Keywords:** Voxel-based morphometry, Cluster headache, Chronic pain, Neuronal plasticity

## Abstract

Cluster headache (CH) is characterized by recurrent episodes of excruciatingly painful, unilateral headache attacks typically accompanied by trigeminal autonomic symptoms. Due to its rhythm with alternating episodes of pain and no-pain, it is an excellent model to investigate whether structural brain changes detected by magnetic resonance based voxel-based-morphometry (VBM) reflect the cause of the disease, may be a consequence of the underlying disease other than pain, or may simply be caused by the sensation of pain itself. We investigated 91 patients with CH in different stages of their disease using VBM and compared them to 78 age- and gender-matched healthy controls. We detected distinct regional gray matter (GM) changes in different brain regions including the temporal lobe, the hippocampus, the insular cortex and the cerebellum. The extent, location and direction of observed GM alterations depended on the state of disease and appeared dynamic in relation to pain state (i.e., pain vs. no-pain). No hypothalamic changes were detected in CH patients compared to healthy controls. The GM changes observed in this study are highly dynamic and thereby reflect the cortical plasticity of the brain in regard to pain. This observed dynamic may provide an explanation of the diverse results of previous VBM studies in pain. Regarding CH the results suggest that the disease is more likely to be caused by a network dysfunction rather than by a single malfunctioning structure.

## Highlights

• We identified gray matter (GM) changes in pain processing areas and beyond in patients suffering cluster headache (CH) compared to healthy controls
• GM changes significantly differed for different courses of the disease (chronic, episodic inside bout, episodic outside bout).
• GM decrease was predominantly observed in chronic CH, while episodic CH showed a more complex and partly opposite behavior.
• This dynamic probably reflects the brain's adaptation capacity to changing stimuli in regard to cortical plasticity and may provide an explanation of the diverse results of previous VBM studies in pain

## 1 Introduction

In contrast to the former belief of a static adult brain without structural changes past full development, considerable plasticity of the adult brain has been well described now. This not only specifically applies to changes caused by training and learning, but also was shown for many other external influences. In regard to pain and headache, numerous studies showed structural brain changes in different conditions that were reversible in parallel to the cessation of pain (Obermann et al., 2009; Rodriguez-Raecke et al., 2009; Gwilym et al., 2010). In experimentally induced pain, structural changes most likely reflect alterations caused by the noxious input, while in disorders like chronic headache the question of cause or consequence of pain and disease is much more difficult to answer.

Cluster headache (CH) as primary headache disorder with strict circannual and circadian rhythm of headache attacks and symptom free episodes is a promising model condition to differentiate structural brain changes primarily related to the headache disorder itself from changes caused by the sensation of pain in general. There are three different stages of disease in CH: 1) episodic CH (eCH) in bout (i.b.) with acute pain attacks up to eight times a day, 2) episodic CH out of bout (o.b.) — an attack free phase that may last months to years, and 3) chronic CH (cCH) without attack free remission periods lasting beyond 1 month. Approximately 10–20% of all CH patients suffer from cCH (Headache Classification Committee of the International Headache Society, 1988).

The clinical characteristics of CH with trigeminal autonomic symptoms (i.e., lacrimation, conjunctival injection, tearing, facial sweating, nasal congestion, miosis and ptosis) as well as the circadian rhythm suggest involvement of the hypothalamus. This involvement was confirmed in several functional imaging studies (May et al., 1998; Sprenger et al., 2004; Morelli et al., 2009). An early voxel-based morphometry (VBM) study detected an isolated regional gray matter increase in the posterior hypothalamus which was thought to be responsible for the development of CH (May, et al, 1999). However, this pathognomonic pathophysiological connection became more and more disputed recently as many other primary headache disorders and different painful conditions showed hypothalamic involvement in imaging studies and newer VBM studies were not able to reconfirm structural hypothalamic alterations in CH (Denuelle et al., 2007; Holle et al., 2011; Kupers et al., 2000; Rosen et al., 1994; Blankstein et al., 2010; Matharu, 2006; Absinta et al., 2012; Yang et al., 2013). It was suggested that the hypothalamus might be unspecific and simply a part of the pain modulating network (Tracey and Mantyh, 2007; Holle et al., 2011).

Since not all studies on different painful disorders were able to show changes in all structures that presumably take part in human pain processing, it remains unclear which structural changes may be caused by the disease itself, which are related to pain in general, and which are a consequence of the underlying disease other than the sensation of pain (Iannetti and Mouraux, 2010).

In this study we used magnetic resonance imaging (MRI) based VBM to 1.) identify different GM change patterns corresponding to different stages of disease in order to differentiate GM changes associated with CH in general from changes related to the sensation of pain itself and 2.) reconfirm the presence of structural GM changes in the hypothalamus and other brain regions known to be associated with trigeminal pain processing.

## 2 Material and methods

### 2.1 Subjects

Ninety-seven patients (75 men, 22 women) with CH were investigated. Clinical characteristics and demography of the ninety-one subjects included into the final analysis are shown in Table 1. Patients were recruited from a tertiary headache center (West-German Headache Center) between April 2009 and August 2011. The study protocol was approved by the local ethics committee and all participants gave their written informed consent according to the Declaration of Helsinki prior to study inclusion. The diagnosis was re-confirmed in a face-to-face interview by headache experienced neurologists (D.H., M.O.) according to the International Classification of Headache Disorders (ICDH-II) (Headache Classification Committee of the International Headache Society, 2004). Inclusion criteria were age over 18 years and confirmed diagnosis of CH. Exclusion criteria were other primary headaches, psychiatric co-morbidities, and other serious somatic illnesses and pain conditions. Patients were compared to 78 healthy age- and gender-matched controls (56 males, 22 females). All subjects included were interviewed using a standardized questionnaire.

### 2.2 Statistical analysis of clinical and demographic data

ANOVA with post-hoc Bonferroni analysis using a cutoff significance level of *p* < 0.05 was performed for clinical data, demographics, estimated volumes of different brain tissue classes (using http://www.cs.ucl.ac.uk/staff/g.ridgway/vbm/get_totals.m) and total intracranial volume (TIV, sum of CSF, gray matter, and white matter) using IBM SPSS Statistics Version 19 (International Business Machines Corporation, Armonk, New York, USA).

### 2.3 VBM — data acquisition, processing and analysis

Imaging of all patients and controls was performed on a 1.5 Tesla scanner (Magnetom Avanto, Siemens Healthcare, Erlangen, Germany) using a standard 8-channel birdcage head coil. No participant was scanned twice. No longitudinal analysis was performed. Prior to analysis all images were rated regarding image quality and pathologies. These were double-checked by an experienced neuro-radiologist (N.T.) blinded to diagnosis and found to be unremarkable in all patients and controls included in the final analysis. T1-weighted magnetic resonance imaging (MRI) 3D datasets were obtained using a magnetization prepared rapid acquisition gradient echo (MP-RAGE) sequence (TR: 2400 ms, TE: 3.52 ms, TI: 1200 ms, flip angle: 8, matrix: 256 × 256 mm², 160 slices, resolution: 1 × 1 × 1 mm³).

Data processing and analysis were performed using SPM8 (Wellcome Trust Centre for Neuroimaging, UCL, London, UK [http://www.fil.ion.ucl.ac.uk/spm/]) including “New Segment”, “DARTEL” (Ashburner, 2007) and MATLAB (MATLAB 7.6.0.324, R2008a, The MathWorks, Natick, MA, USA). Preprocessing involved “unified segmentation” (incl. normalization into the Montreal Neurological Institute (MNI) space) and modulation in order to adjust for volume changes during spatial normalization (Wright et al., 1995; Ashburner and Friston, 1997; Friston, 1995; Good et al., 2001). Spatial smoothing was performed with an isotropic Gaussian kernel of 10 mm full-width at half maximum (Ashburner and Friston, 2005). Prior to preprocessing images of patients suffering from left-sided CH were flipped to enhance analysis. Additionally, unflipped analysis was performed to avoid false positive results due to normal brain asymmetry. It showed alterations in the same brain regions, but observed effect strengths were lower. Statistical whole brain analysis tested GM volume differences between CH patients and healthy controls (HC). Post-hoc subgroup analysis was performed comparing the following groups with healthy controls: (1) episodic CH i.b., (2) episodic CH o.b., and (3) cCH. Although gender and age matching was performed these factors were also included into the statistical model along with total intracranial volume. Gray matter changes are reported with a threshold of *p*_FWE_ < 0.05 and correction for multiple comparison (family wise error). To avoid unintentional bias by a priori hypothesis, have better comparability to previous pain VBM studies, and not miss false negative regions a threshold of *p*_unc_ < 0.001 uncorrected and a voxel size greater than 30 voxels were also investigated.

## 3 Results

### 3.1 Clinical characteristics and demographics

Table 1 summarizes the clinical characteristics and demographics of study participants. Physical and neurological examination was unremarkable in all patients and controls. Controls did not suffer from any headache or other psychiatric or severe somatic disorder. No differences between the groups were observed in regard to age, gender, disease duration, attack frequency, average attack duration or number of attacks per day during active periods (i.e., eCH i.b. and cCH). Patients were categorized to be out of bout when 15 headache free days were reached. No patient suffered a headache attack during the scanning procedure. Patients currently not suffering from headaches (eCH o.b.) remembered and rated their pain higher on a verbal analogue scale than cCH patients (eCH o.b.: 9.326 ± 0.94; cCH: 8.217 ± 1.54).

### 3.2 Voxel-based morphometry

**Fig. 1:**
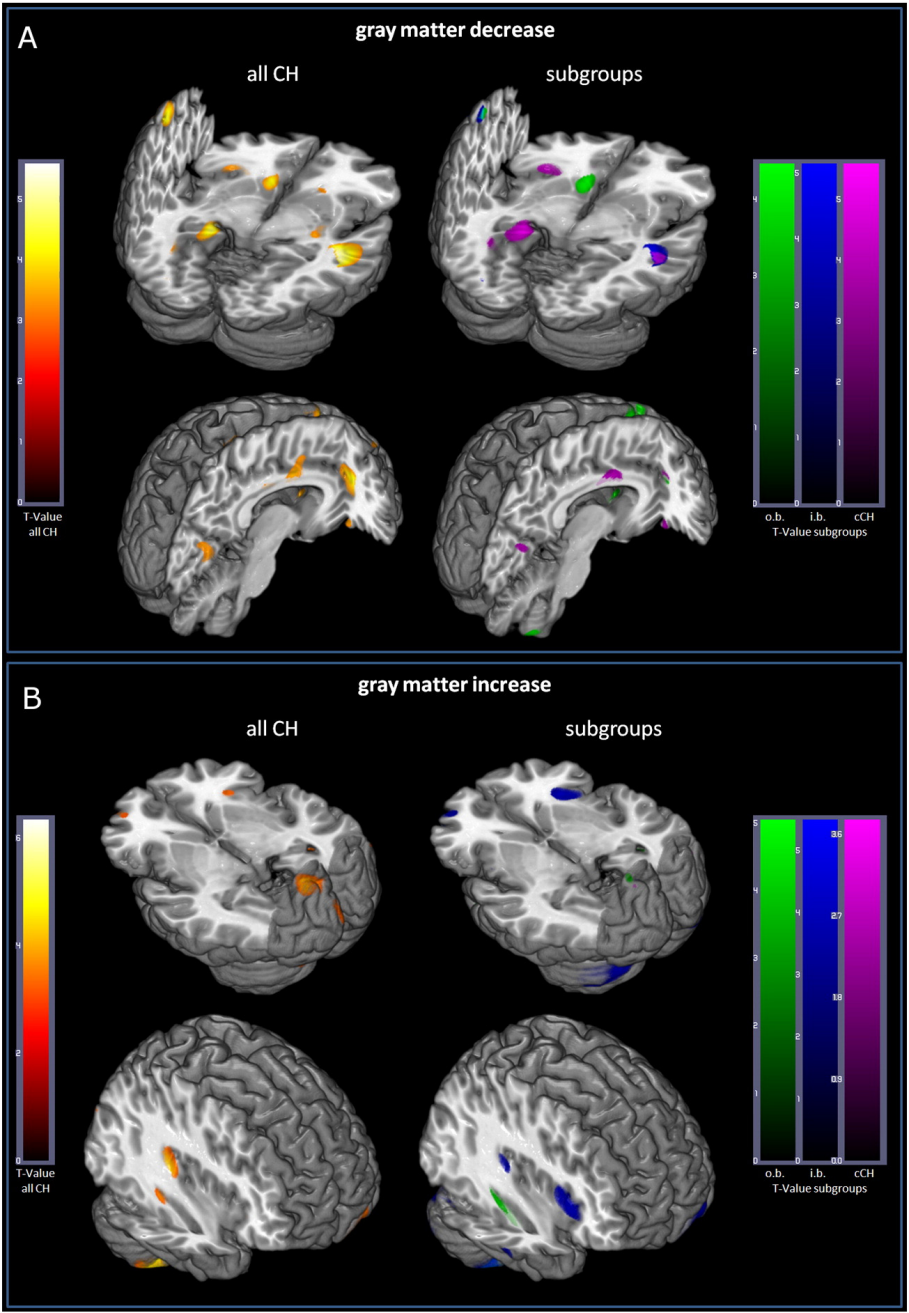
Gray matter changes in cluster headache. Areas with significant GM decrease (A) or increase (B) in overall comparison (left, CH vs. HC, *p*_unc_ < 0.001) and subgroup analysis (right; episodic CH outside bout (green), inside bout (blue), and chronic CH (purple), CH vs. HC, *p*_unc_ < 0.001). A: Areas with corresponding GM decrease from (left to right): first row: primary sensory cortex (S1), left inf. temporal gyrus, left dorsal hippocampus, left anterior insula, left caudate ncl, right caudate nlc., and right mid. temporal lobe; second row: right occipital lobe, right posterior ACC, left superior medial gyrus, right perigenual ACC, and right orbitofrontal cortex. MNI-coordinates, *T*-value, and k_E_ (cluster-size) are given in Tables 3 and S1. B: Areas with corresponding GM increase from (left to right): first row: left orbitofrontal cortex, right anterior insula, left occipital lobe, right ventral hippocampus, and left area 17; second row: right cerebellum, right ventral hippocampus, right posterior insula, (right anterior insula; overall not visible in this slice), and left orbitofrontal cortex. MNI-coordinates, *T*-value, and k_E_ (cluster-size) are given in Tables 3 and S2.

**Fig. 2:**
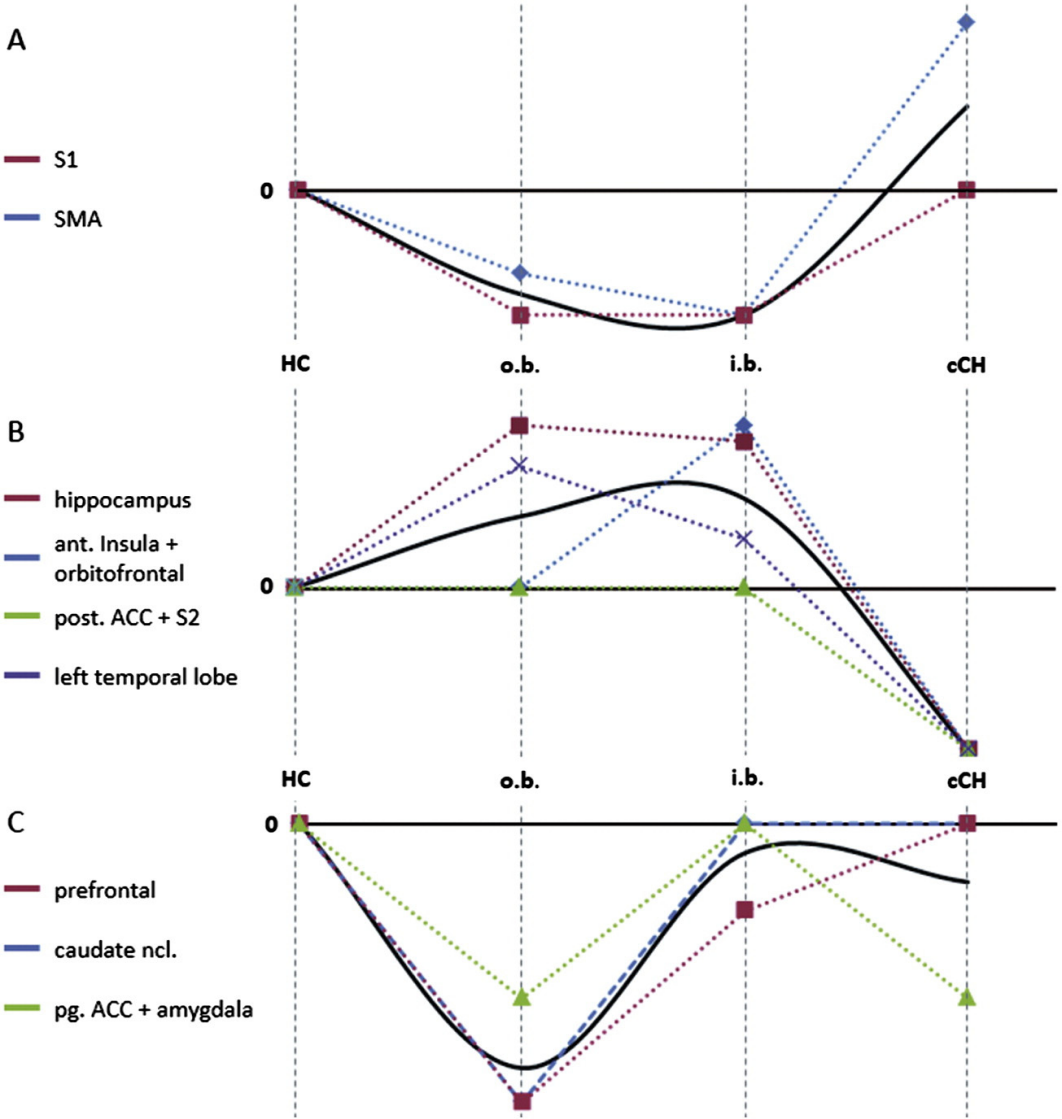
Gray matter alteration in different brain areas in the different states of CH. Schematic overview over different areas with similar GM change behavior over different disease states. Colored lines reflecting region-wise GM alteration as indicated on the left. Black lines show the estimated/approximated pattern dynamic. A: Areas with predominant GM loss in episodic subtypes (i.b.) with GM increase and normalization in chronic CH (S1 = primary somatosensory cortex, SMA = supplementary motor cortex) representing an episodic or transient pain disease pattern. B: Areas with GM decrease in cCH, with increase in eCH (i.b. > o.b) representing a chronic pain disease pattern. C: Areas with pronounced GM decrease in pain free state (o.b.).

Seven scans (5 patients, 2 controls) were excluded from the final analysis due to movement artifacts. Another patient was excluded due to previously unknown metal splint artifacts. In the remaining 169 (91 patients + 78 controls) MRIs included into the final analysis no morphological abnormalities or artifacts were observed on visual inspection of T1 weighted images. The results of the overall analysis comparing all CH patients with healthy controls are displayed in Fig. 1 and Tables 2 and 3. In different areas similar GM changes over different subgroups were detected. This is schematically illustrated in Fig. 2. ANOVA for volumes of different brain tissue classes revealed significant GM volume reduction in chronic CH compared to other patient subgroups. White matter, CSF or total intracranial volume did not differ between groups.

#### 3.2.1 All CH patients vs. healthy controls

##### 3.2.1.1 GM decrease comparing all CH patients with healthy controls

CH patients showed GM decrease in the ipsilateral temporal lobe and contralateral dorsal hippocampus after correcting for multiple comparisons (*p*_FWE_) *p* < 0.05. Below an uncorrected threshold of *p*_unc_ < 0.001 GM volume decrease in pain processing structures such as the anterior cingulate cortex (ACC), striatum, orbitofrontal cortex, hippocampus, amygdala, insular cortex, and primary (S1) and secondary (S2) somatosensory cortices was detected as well as in the contralateral temporal lobe, and premotor and occipital cortices (Tables 2 and 3).

##### 3.2.1.2 Gray matter increase comparing all CH patients with healthy controls

GM increase in CH could also be identified. The changes were most pronounced in the cerebellum bilaterally and the ipsilateral posterior insula passing a corrected threshold for multiple comparisons of *p*_FWE_ < 0.05. Alterations in the temporal lobe including the hippocampus, as well in the occipital lobe and the anterior insular cortex were below the threshold of *p*_unc_ < 0.001 uncorrected (Tables 2 and 3).

#### 3.2.2 Subgroup analyses of CH patient's o.b., i.b., and cCH vs. HC

Table 2 gives an overview of the results of overall and post hoc subgroup analyses. Comparing the alterations of the subgroups vs. HC to each other it becomes obvious that GM alterations differ substantially within the different stages of disease. Some areas involved in pain processing showed even opposite behavior. For example in cCH most areas showed a GM decrease while in some increase was seen in eCH inside bout. Cluster headache patients out of the bout in general showed less marked GM alterations compared to the other groups, but some of those were quite characteristic (e.g. decrease in the striatum and prefrontal areas). Details on subgroup analyses are given in the supplement (Tables S1 and S2).

#### 3.2.3 Intergroup analysis

Comparing different patient subgroups with each other showed corresponding GM changes to overall and subgroup analyses which are given in the supplement Table S3. To put it in a nutshell, with only two exceptions i.b. patients showed GM increase while cCH showed GM decrease in comparison to the other subtypes.

#### 3.2.4 Correlation-analysis

**Fig. 3:**
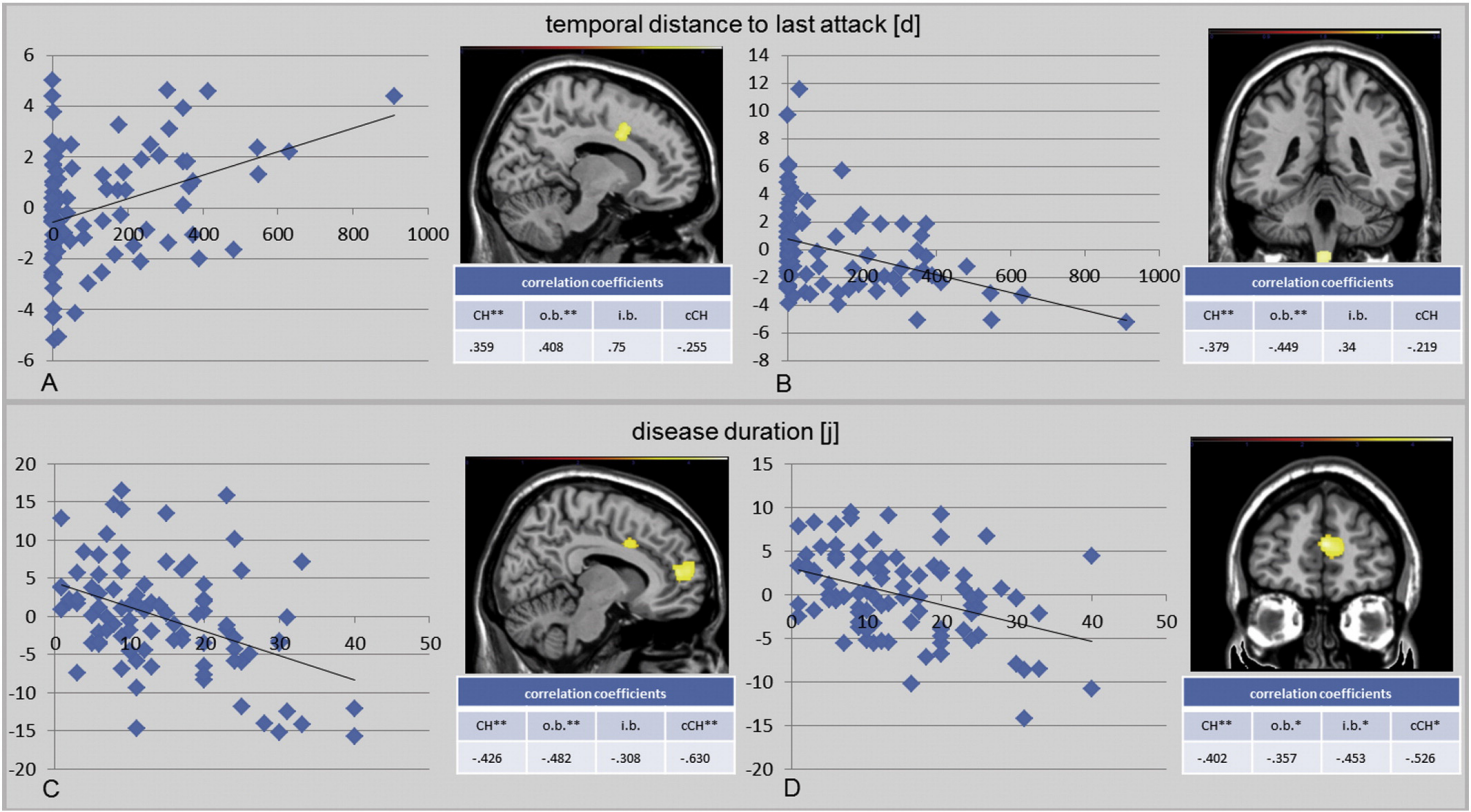
Gray matter correlation analysis. Correlation analysis: areas with significant GM increase or loss in correlation with days gone since last attack (A + B) and disease duration (C + D), left-sided graphical correlation, right-sided GM changes in image and correlation coefficient. Significance level: *p < 0.05; **p < 0.01, not starred = not significant. MNI coordinates and details on overall correlation: A + B: pACC (x: 15 y: 6 z: 33; k_E_: 431; T: 4.31), brainstem (x: 2 y: 36 z: 66; k_E_:468; T: 3.64); C + D: pACC (x: 8 y: 9 z: 37; k_E_: 192; T: 3.61); prefrontal cortex (x: 3 y: 51 z: 13; k_E_:1113; T: 4.69).

Correlation analysis was performed for disease duration, attack frequency and the number of days since the last attack for all CH patients compared to HC and post-hoc analysis was performed for each patient subgroup (Fig. 3).

For the number of days since the last attack, GM volume showed a positive correlation (GM increase) in the posterior ACC. A GM decrease in parallel with the number of days since the last attack in the brainstem/spinal trigeminal nucleus was detected in overall correlation. In subgroup analysis these effects were only significant in patients currently o.b.

In regard to disease duration the posterior portion of the ACC showed GM decrease in overall comparison, which was mainly related to the cCH subgroup and remained significant in patients outside bout as well. Prefrontal GM decrease in correlation to disease duration was seen for all groups. No correlation was found for attack frequency.

## 4 Discussion

Characteristic distinct regional GM volume changes were seen in different states of disease in patients with CH. Most of the observed changes are comparable to previously described findings. By virtue of the episodic course of CH (i.e., transition of attack rich and attack free states), we were able to further differentiate GM changes related to transient (i.b.) and chronic (cCH) pain attacks, as well as changes possibly related to the disease itself (i.e., in CH o.b.) — either as a consequence of it or as a part of its underlying origin.

### 4.1 Main findings

The following discusses the brain areas with GM changes that survived multiple comparison testing at a significance threshold of *p* < 0.05 (FWE). Additional considerations will also acknowledge findings with a lower, uncorrected significance threshold of *p* < 0.001 (see Section 4.3) to be able to better compare our findings to previous studies on pain processing.

Alterations detected in the temporal lobe were most pronounced, but partly divergent comparing different states of CH. The right middle temporal lobe showed GM decrease in all analyses. This was FWE-significant in all CH and i.b. patients only. Functional and structural (Holle et al., 2011; Rocca et al., 2006; Schmidt-Wilcke et al., 2007; Schmidt-Wilcke et al., 2005) alterations in the temporal lobe were often described in different painful conditions, but its role for central pain processing remains unclear and may be underrated. As the medial temporal lobe is associated with the attentional and emotional modulation of pain perception (Ploner et al., 2011), the role of the lateral temporal lobe remains uncertain. In patients with migraine, increased excitability of the temporal lobe was interpreted as part of central sensitization that is well known for migraine (Moulton et al., 2011). This would fit our finding of inferior temporal lobe GM decrease in chronic CH quite well, if central sensitization could be regarded as one important step towards the development of chronic pain as suggested previously (Obermann et al., 2009).

The second structure with significant alterations in FWE corrected analysis and antipodal behavior in the different states of CH is the insular cortex. The right posterior insular shows FWE significant GM increase in all CH patients and patients inside bout. The insula is well known to show GM alterations in different pain conditions (May, 2011). Functional imaging (Peyron et al., 2000) revealed that it codes for many functions in regard to pain perception and processing including the modulation of pain in a pro- and anti-nociceptive manner (Jasmin et al., 2003). Particularly this could provide a valid explanation for the structural reorganization within this region from episodic pain to cCH. From our data, GM increase within the anterior insula seems to be predominantly related to patients currently inside bout, while GM decrease was found predominantly in cCH. The posterior insula on the other hand shows GM increase over all subgroups (most pronounced in patients inside bout). In regard to pain processing, the insula may consist of two different functional parts as the anterior insula shows the described dichotomic reorganization behavior from transient to chronic pain, while the posterior insula does not. The different parts are believed to have different functions in regard to sensation, emotion and behavior (Ploner et al., 2011; Bernhardt and Singer, 2012; McGlone et al., 2012; Gerstner et al., 2012). In pain, as for other senses, the insula's integrative capacities may be enormous, which could explain the rather complex behavior of this structure to changing neuronal input that certainly requires further research before it can even be remotely understood.

GM in the left dorsal hippocampus was altered in overall comparison and in patients suffering from the chronic subtype. The ventral part of this anatomic-structure behaved exactly the opposite way in showing GM increase (uncorrected) in transient pain. The ventral hippocampus is associated with fear conditioning and therefore most likely much more associated with acute pain (González-Pardo et al., 2012). The dorsal hippocampus is associated with learning of conceptual information, which could be considered closer related to a continuing experience of pain (Fanselow and Dong, 2010). Even more interesting is the transition from GM increase in recently developed pain (eCH) within the ventral hippocampus to decrease within the dorsal hippocampus in cCH. This nicely fits the observation that an increase is mostly associated with acute pain, while a decrease is more related to a chronic condition. A number of other brain regions such as the orbitofrontal and anterior insula show a similar structural reorganization and change from GM increase in eCH i.b. to a GM decrease in cCH (Fig. 2). This structural brain plasticity was previously described in longitudinal VBM studies and appears to be closely related to functional changes (Obermann et al., 2009; Rodriguez-Raecke et al., 2009; Gwilym et al., 2010; Teutsch et al., 2008).

The cerebellum showed significant GM increase over all analyzed patient cohorts. When correction for multiple comparisons was applied observed alterations remained significant except in cCH. Although cerebellar involvement in pain processing was shown very early using anatomical (Randić et al., 1981), electrophysiological (Jie and Pei-Xi, 1992) and functional MR-imaging (Helmchen et al., 2004; Helmchen et al., 2003) techniques very little is known about its role in pain. Since most imaging studies investigating pain do not use specific cerebellar normalization procedures imaging results have to be interpreted very carefully. Current discussion regarding cerebellar function in pain processing includes direct nociceptive encoding and modulation, affective processing, sensory–motor integration, withdrawal response and even anticipation (Moulton et al., 2010). More research is needed to further illuminate this.

### 4.2 Hypothalamus

The first VBM study on CH detected isolated GM increased in the inferior posterior hypothalamus compared to HC (May et al., 1999). More recent VBM studies did not find structural GM changes in the hypothalamus (Matharu, 2006; Absinta et al., 2012; Yang et al., 2013). This also accounts for our study. Increasing evidence for an unspecific role of the hypothalamus in pain processing was gathered during the past years where structural and functional data in different conditions were able to show hypothalamic involvement (Holle et al., 2011). The hypothalamus is involved in many regulatory mechanisms including pain processing, but appears not to be specific for CH.

### 4.3 Additional considerations

#### 4.3.1 Episodic cluster headache inside bout

Regional GM changes (mostly GM-increase, otherwise indicated as decrease) related to acute and transient pain in patients with episodic CH i.b. were most pronounced in the primary somatosensory cortex (S1, GM-decrease), anterior and posterior insula, ventral hippocampus, orbitofrontal gyrus, supplementary motor area (SMA, GM-decrease), cerebellum, and occipital cortex (Figs. 1 and 2). This is in line with the current literature that concedes acute pain to be associated predominately with primary and early secondary sensory processing areas (i.e., S1, insula), motor processing areas (i.e., SMA, cerebellum), as well as areas related to cognition and emotional processing (i.e., hippocampus, orbitofrontal cortex) (Rocca et al., 2006; Kim et al., 2008; Schmidt-Wilcke et al., 2008). The role of the occipital cortex remains unclear. Even though it is often described in different pain conditions a good explanation of its function in pain processing is still missing.

Most altered brain regions identified in patients i.b. showed GM increase except for S1, SMA and the middle temporal gyrus. GM increase is considered to be associated with acute and repeated painful stimuli. Teutsch et al. for example showed GM increase in pain processing areas after repeated painful stimuli over several days (Teutsch et al., 2008). This is consistent with VBM data for exercise and learning and the concept of activation dependent brain plasticity demonstrated in humans (Draganski et al., 2004; Boyke et al., 2008). Absinta et al. showed regional GM increase in eCH (Absinta et al., 2012) and Maleki et al. demonstrated an increase in basal ganglia in high frequent migraine (Maleki et al., 2011). Interestingly, a very recent, but small longitudinal study in CH showed GM increase in bout-state compared to o.b. in some regions including the cingulate and insular cortex (Yang et al., 2013). A possible explanation for GM increase in acute pain may be that the brain only reacts with local GM increase until a particular task/stimulus is learned or processed adequately and further on recedes (May, 2009). Regarding acute pain as part of the experience of learning is further supported by our finding in the hippocampus. Interestingly, the insular cortex showed similar behavior. Both regions are discussed in more detail above.

Another region showing an increase in acute and decrease in chronic CH is the orbitofrontal cortex. As part of the prefrontal cortex, it was often described in different VBM studies on pain with clear association to the affective component of pain in general and to the development of chronic pain in particular (Schmidt-Wilcke et al., 2005; Kim et al., 2008; Younger et al., 2010). Its main role is sensory integration, decision-making and expectation (Kringelbach, 2005) and it is involved in the expectation of a reward or punishment for any particular action (Schoenbaum et al., 2011). This may explain its role for pain processing.

The primary somatosensory cortex (S1) and SMA behave opposite to most other brain areas altered in acute pain. Contrary to our current understanding and our hypothesis, S1 shows GM decrease in acute pain and persistent GM decrease in patients out of bout, possibly the primary processing structures are more sensible to recurrent input and change more quickly to repeated painful stimuli as adaptive mechanism possibly reflecting habituation.

#### 4.3.2 Chronic cluster headache (long standing pain-attacks)

Chronic CH patients predominantly showed GM decrease compared to HC. This was reconfirmed by intergroup analysis. The decrease was mainly detected in higher order, secondary and integrative cortical processing centers such as the secondary somatosensory cortex (S2), the posterior part (pACC) and the perigenual portion (pgACC) of the anterior cingulate cortex, the amygdala and in the above discussed regions orbitofrontal cortex, hippocampus, insular cortex and the inferior temporal lobe (Fig. 2B). These regions are well known to be associated with human pain processing and were consistently described with GM decrease in the current literature (see reviews (Tracey and Mantyh, 2007; May, 2011; May, 2009)). Several of these regions were proposed to be especially important for the development and maintenance of chronic pain in particular, such as the pACC and the amygdala (Obermann et al., 2009; Rodriguez-Raecke et al., 2009; Gwilym et al., 2010). Our data reconfirm this connection to chronic pain and underline once again the neuroplastic capacity of these brain regions in response to changing stimuli. GM decrease was detected in cCH within the pACC and GM volume correlated with the interval between attacks, which was mainly driven by CH outside bout. This could point towards a recovery of this area after the painful episode recedes. No significant correlation in this regard was found in the patients suffering from cCH. It appears that the recovery potential of this area is severely impaired in patients with longstanding pain-attacks. Similar, hypothetically reactive increases of GM were described in longitudinal studies in regard to cortical plasticity of the pACC (Obermann et al., 2009; Rodriguez-Raecke et al., 2009). The incapacity of pACC recovery in chronic pain is further supported by a negative correlation of GM volume with disease duration. The prefrontal cortex, another potentially anti-nociceptive region, behaved similarly.

GM decrease was also seen in the amygdala and pgACC in patients with cCH confirming their role in chronification of pain. However, comparable changes were detected in CH o.b., which may hint at a predominantly anti-nociceptive mechanism and an active attempt to suppress or prevent the chronification. Extensive connections from the amygdala to the ACC were described, along with their possible role for the defensive behavioral system that controls transmission of nociceptive impulses to the brain through multiple circuits that can be modulated by stress, fear and expectation (Coghill et al., 2001; Sikes and Vogt, 1992; Bingel et al., 2002). The amygdala contributes to emotional processing rather than to sensory discriminative components of pain processing (Bingel et al., 2002) and was demonstrated to show GM decrease in chronic pain due to osteoarthritis that recovered completely within 18 weeks after hip replacement surgery (Rodriguez-Raecke et al., 2009).

As mentioned before hippocampal alterations were of complex behavior. Regional GM decrease in the dorsal hippocampus may suggest a connection of memory and chronic pain. Previous VBM studies of chronic pain reported changes in the hippocampus in support of this assumption (Lutz et al., 2008; Schweinhardt et al., 2008). The negative effect of chronic stress on the plasticity of the hippocampal formation is undisputed. Chronic or persistent recurring pain is a major stressor in this regard and in animal models leads to functional and morphological changes of the hippocampus (McEwen, 2001). The reduction of GM volume in cCH patients supports the association of hippocampus affection and chronification.

#### 4.3.3 Pain-free state (out of bout)

Three possible mechanisms responsible for the underlying GM changes o.b. must be considered. Changes may be residual alterations persisting after the last bout, changes may represent a predisposition for CH, or they may reflect an effective anti-nociceptive network performance.

Since the areas altered most distinct in patients outside bout (prefrontal cortex and caudate nucleus), do not show marked alterations in the other subgroups they are not likely to be “left over” and so have to be evaluated in the context of predisposition or anti-nociception. Prefrontal cortico-striatal connections were just recently described to correlate with the persistence of pain (Baliki et al., 2012). The persisting GM decrease may suggest a dysfunction or incomplete connectivity between the prefrontal cortex and the striatum in CH. This hypothesis is further supported by FDG-PET data showing hypometabolism in frontal brain areas and caudate nucleus predominantly in CH patients out of bout (Sprenger et al., 2007). Involvement of the prefrontal cortex and in CH was also described in two recent VBM analyses and was interpreted as a possible correlate of an impaired descending pain modulation system (Absinta et al., 2012; Yang et al., 2013). This underlines our explanation of a deficient pain processing network leading to pain in CH (Sprenger et al., 2007).

### 4.4 Methodological considerations

The core strength of this study is the large patient numbers that allow sufficient subgroup analysis compared to properly matched healthy controls. This is unique in the investigation of primary headache disorders and provides insight into the dynamic of described GM changes in regard to the sensation of pain. One of the main limitations of this study is the cross-sectional design, which not only gives a first impression of the dynamic of underlying GM changes following pain but also demonstrates the urgent need for longitudinal analyses. Unfortunately, VBM still has considerable methodological limitations. It remains unclear whether GM alterations are caused by irreversible mechanisms such as neuronal degeneration/apoptosis, fast adjusting reversible neuronal processes such as dendrite spine and synapse turnover, or whether they simply reflect changes in extracellular space, microvascular volume or blood flow (Apkarian et al., 2004; Franklin et al., 2013).

The experience of pain has a powerful influence on psychology. It is common sense that receptive or permanent painful stimuli have major impact on mood and anxiety. The comorbidity of headache, depression and anxiety is well investigated (Mercante et al., 2011; Antonaci et al., 2011; Breslau et al., 2003; Mitsikostas and Thomas, 1999; Juang et al., 2000) and probably shares common central processing pathways (Milham et al., 2005; Radua et al., 2010; Yoo et al., 2005).

Another point to mention is the influence of preventive medication. Most of the patients were on corticosteroids, verapamil and/or topiramate. Only some were on lithium. No proper data exists for VBM with verapamil or topiramate. Lithium was studied in small groups of healthy humans and tends to cause a GM increase but studies are not consistent regarding location (Cousins et al., 2013; Monkul et al., 2007). The influence of depression, anxiety or medication cannot be ruled out. One could argue that the time window for classification of episodic patients (15 days) may be too tight or too lose. Our prospective decision to use 15 days as cutoff was chosen as a good balance between possible misclassification of patients almost out of the bout and already receding neuroplastic changes on the other hand.

## 5 Conclusion

In conclusion, characteristic GM change patterns for different stages of disease in CH are highly dynamic and reflect the brain's adaptation capacity to different stimuli in regard to cortical plasticity. It is worth noticing that GM decrease is predominantly seen in chronic pain, while acute pain shows a more complex and partly opposite behavior. This dynamic provides an explanation for the diverse results of previous VBM studies in pain. Different brain regions seem to have different adaptation capacities and react differently to changing pain stimuli. Moreover the brain apparently utilizes different parts of the same system for the procession of acute and chronic pain. It becomes evident that complex and remittent recurrent diseases such as CH are most likely related to dysfunctional nociceptive and anti-nociceptive processing networks. Disturbed inhibition and facilitation mechanisms may lead to the development of recurrent painful attacks as well as chronic pain in some patients eventually.

## Conflict of interest statement

There is no potential conflict of interest regarding this study.

## Figures and Tables

**Table 1:** Demographics and clinical characteristics of different cohorts and subgroups.

	HC	eCH o.b.	eCH i.b.	cCH	CH	p/F
Group size	78	46	22	23	91	
Age [years]	42.78 ± 11.44 [18–64]	44.35 ± 10.95 [18–67]	45.41 ± 9.60 [28–67]	47.96 ± 10.56 [23–65]	45.52 ± 10.61 [18–67]	0.241/1.413
Men/women	56/22	36/10	19/3	16/7	71/20	0.468/0.851
Number of attacks/day	–	3.42 ± 2.34 [1–8]	2.68 ± 1.49 [1–6]	2.57 ± 1.92 [0.5–7]	3.03 ± 2.08 [0.5–8]	0.145/1.973
Last attack [days]	–	241.20 ± 186.83 [16–911]	2.82 ± 4.03 [0–14]	2.39 ± 3.64 [0–12]	123.21 ± 178.46 [0–911]	<0.001/36.260
Duration of disease [years]	–	16.89 ± 9.64 [1–40]	11.73 ± 9.55 [1–33]	12.09 ± 7.63 [2–30]	14.42 ± 9.39 [1–40]	0.039/3.373
Av. attack duration [min]	–	81.63 ± 59.55 [15–180]	55.68 ± 43.60 [15–180]	55.21 ± 33.12 [15–180]	68.68 ± 52.58 [15–180]	0.067/2.794
Total intracranial volume [ml]	1625.04 ± 160.11 [1274.74–1958.03]	1639.53 ± 126.12 [1322.56–1857.49]	1660.43 ± 135.63 [1409.22–1958.30]	1561.27 ± 155.06 [1274.74–1840.63]	1624.80 ± 139.91 [1274.74–1958.3]	0.177/1.994

HC = healthy controls; eCH o.b. = episodic cluster headache outside bout; eCH i.b. = episodic cluster headache inside bout; cCH = chronic cluster headache, CH = all cluster headache patients.

**Table 2:** Overview of gray matter changes in cluster headache.

Areas with GM changes in overall comparison		All CH vs. HC	Overview subgroup		
			eCHob	eCHib	cCH
a. Significant alterations using *p*_FWE_ < 0.05					
Temporal	Right mid. temporal gyrus	↓*	↓	↓↓*	↓
Hippocampus	Left dorsal hippocampus	↓*			↓↓*
Insula	Right posterior insula	↑*	↑	↑↑*	↑
Cerebellum	Right cerebellum	↑*	↑↑*	↑↑*	↑↑
	Bilateral cerebellum	↑*		↑↑*	
b. Significant alterations using *p*_unc._ < 0.001					
Temporal	Left inferior temporal gyrus	↓	↑		↓↓
	Left middle temporal gyrus	↑	↑↑	↑	
Hippocampus	Right ventral hippocampus	↑	↑↑	↑↑	
	Left ventral hippocampus	↑	↑	↑↑	
Amygdala	Right amygdala	↓	↓		↓
Insula	Left anterior insula	↓			↓↓
	Right anterior insula	↑		↑↑	
Basal ganglia	Left caudate nucleus	↓	↓↓		
	Right caudate nucleus	↓			
Orbitofrontal	Right orbitofrontal cortex	↓			↓↓
	Left orbitofrontal cortex	↑		↑↑	↓
Pre-frontal	Right superior frontal gyrus	↓			
	Left superior medial gyrus	↓	↓↓	↓	
	Left superior frontal gyrus	↓	↓↓		
SMA	Left SMA/area 6	↓	↓	↓↓	
	Right SMA/area 6	↑		↓	↑↑
Somato-sensory	Left primary somatosensory cortex	↓	↓	↓	
	Right secondary somatosensory cortex	↓			↓↓
Cingulate	Right perigenual ACC	↓	↓		↓
	Right posterior ACC	↓			↓↓
Occipital	Right occipital lobe	↓			↓↓
	Left occipital lobe	↑	↑	↑	↑
	Area 17	↑			↑↑

Areas with significant GM changes (decrease or increase as marked by arrows) in overall comparison (CH vs. HC, *p*_unc_ < 0.001, *p_FWE_ < 0.05 threshold > 30 voxels) and overview of corresponding GM alterations in subgroup analysis. right-sided = ipsilateral, left-sided = contralateral to headache; ↓↓/↑↑ = strong GM change in subgroup analysis (more than 90% of *T* value or cluster extent in comparison to overall analysis). MNI-coordinates, *T*-value, and k_E_(cluster-size) are given in Tables 3 and S1 (GM-decrease) and S2 (GM-increase).

**Table 3:** Detail information on GM changes (decrease and increase) in overall comparison (CH vs. HC).

	MNI coordinates			T	k_E_
	X	Y	Z		
	GM-decrease				
Right mid. temp. gyrus*	47	−31	−3	5.59	1905
Left inferior temporal gyrus	−36	−46	−12	4.04	454
Left caudate ncl.	−11	23	−8	4.36	244
Right caudate ncl	11	24	9	4.18	239
Right orbitofrontal cortex	12	44	−21	3.53	86
Right superior frontal gyrus	20	56	36	3.52	144
Left superior medial gyrus	−2	33	60	3.99	191
Left superior frontal gyrus	−12	33	40	3.98	334
Left SMA /area 6	−18	−7	57	4.29	301
Left primary somatosensory cortex	−53	−19	49	4.43	616
Right secondary somatosensory cortex	53	−45	25	3.64	129
Left dorsal hippocampus*	−23	−38	−3	5.08	358
Left ant. insula	−24	26	−2	4.04	206
Right amygdala	35	−4	−15	4.39	294
Right. perigenual ACC	17	41	12	4.37	571
Right posterior ACC	12	6	33	4.27	536
Right occipital cortex	11	−66	3	3.45	191
	GM-increase				
Right posterior insula*	50	−27	15	5.13	2046
Right anterior insula	32	29	−5	3.44	41
Left ventr. hippocampus	−23	−6	−48	4.27	201
Left mid. temporal gyrus	−44	−52	4	4.24	178
Right ventr. hippocampus	36	−36	−11	3.73	297
Left occipital lobe	−18	−58	34	4.52	2280
Area 17	−3	−100	15	3.38	129
Left orbitofrontal	−11	62	−23	3.59	491
Right SMA/area 6	48	−6	51	3.31	37
Right cerebellum*	27	−40	−53	3.34	2236
Bilateral cerebellum*	6	−67	−18	4.86	4539

*p*_unc_ < 0.001; *p_FWE_ < 0.05 threshold > 30 voxels; MNI — Montreal Neurological Institute coordinates, T: effect strength, k_E_: cluster-size; right-sided = ipsilateral, left-sided = contralateral to headache.
