# Supplementary material for: Cortical plasticity in episodic and chronic cluster headache
Source: Neuroimage Clin. 2014 Oct 18;6:415–23. doi: 10.1016/j.nicl.2014.10.003 (PMC4218933; doi:10.1016/j.nicl.2014.10.003)
Supplement: Supplementary file 1 — Supplementary tables. [file mmc1.docx]

**Supplement:**

**Tab. S1: Detail information on subgroup analysis for all areas with significant GM-decrease represented by overall comparison:**

|  | **MNI Coordinates** | | | **T** | **k_E_** |
| --- | --- | --- | --- | --- | --- |
|  | **X** | **Y** | **Z** |  |  |
| **Episodic CH outside bout vs. HC** | | | | | |
| Right mid. temp. gyrus | 47 | -36 | 4 | 3.33 | 69 |
| Left caudate ncl. | -9 | 21 | -6 | 4.47 | 323 |
| Left superior medial gyrus | -6 | 41 | 57 | 3.82 | 282 |
| Left superior frontal gyrus | -12 | 35 | 39 | 3.61 | 79 |
| Left SMA/Area 6 | -8 | -16 | 57 | 3.51 | 50 |
| Left primary somatosensory cortex | -53 | -18 | 49 | 3.45 | 103 |
| Right amygdala | 33 | -4 | -12 | 3.47 | 48 |
| Right. perigenual ACC | 17 | 41 | 12 | 3.28 | 41 |
| **Episodic CH inside bout vs. HC** | | | | | |
| Right mid. temp. gyrus* | 47 | -31 | -3 | 5.14 | 1486 |
| Left superior medial gyrus | -2 | 33 | 60 | 3.59 | 48 |
| Left SMA /Area 6 | -15 | -6 | 58 | 3.84 | 175 |
| Right SMA | 15 | 8 | 46 | 3.6 | 132 |
| Left primary somatosensory cortex | -56 | -21 | 51 | 3.88 | 353 |
| **Chronic CH vs. HC** | | | | | |
| Right mid. temp. gyrus | 47 | -33 | -2 | 3.54 | 250 |
| Left inferior temporal gyrus | -50 | -51 | -23 | 3.96 | 653 |
| Right orbitofrontal cortex | 14 | 42 | -23 | 3.32 | 72 |
| Left orbitofrontal cortex | -24 | 17 | -24 | 3.90 | 319 |
| Right secondary somatosensory cortex | 53 | -46 | 28 | 3.61 | 106 |
| Left dorsal hippocampus* | -15 | -39 | 6 | 5.18 | 845 |
| Left anterior insula | -26 | 26 | -2 | 3.71 | 365 |
| Right amygdala | 33 | -4 | -14 | 3.51 | 80 |
| Right perigenual ACC | 15 | 45 | 15 | 3.4 | 65 |
| Right posterior ACC | 6 | 5 | 25 | 3.98 | 377 |
| Right occipital cortex | 8 | -64 | 13 | 3.31 | 60 |

p_unc_<0.001, *p_FWE_<0.05 Threshold >30Vx; MNI-Montreal Neurological Institute coordinates, T:effectstrength, k_E_:cluster-size; rightsided = ipsilateral, leftsided = contralateral to headache.

**Tab. S2: Detail information on subgroup analysis for all areas with significant GM-increase represented in overall comparison:**

|  | **MNI-Coordinates** | | | **T** | **k_E_** |
| --- | --- | --- | --- | --- | --- |
|  | **X** | **Y** | **Z** |  |  |
| **Episodic CH outside bout vs. HC** | | | | | |
| Left middle temporal Gyrus | -45 | -55 | 3 | 4.64 | 360 |
| Left inferior temporal gyrus | -50 | -7 | -27 | 4.54 | 283 |
| Right post insula | 51 | -25 | 19 | 3.29 | 46 |
| Left ventral hippocampus | -21 | -4 | -45 | 3.37 | 33 |
| Right ventral hippocampus | 35 | -22 | -21 | 4.21 | 389 |
| Left occipital lobe | -20 | -54 | 15 | 3.97 | 623 |
| Right cerebellum* | 26 | -40 | -53 | 5.05 | 1161 |
| **Episodic CH inside bout vs. HC** | | | | | |
| Left mid. Temporal gyrus | -45 | -57 | 4 | 3.47 | 34 |
| Left orbitofrontal cortex | -24 | 60 | -11 | 4.09 | 787 |
| Left ventral Hippocampus | -24 | -4 | -48 | 3.94 | 179 |
| Right ventral hippocampus | 30 | -6 | -36 | 4.03 | 181 |
| Right post insula* | 51 | -16 | 4 | 4.99 | 1476 |
| Right ant. insula | 32 | 27 | -5 | 3.98 | 715 |
| Left occipital lobe | -17 | -82 | 31 | 3.74 | 372 |
| Right cerebellum* | 29 | -39 | -54 | 5.50 | 1167 |
| Bilateral cerebellum* | 6 | -67 | -18 | 5.41 | 5775 |
|  | -24 | -85 | -24 | 4.07 | 2246 |
| **Chronic CH vs. HC** | | | | | |
| Right SMA/Area 6 | 57 | -3 | 51 | 3.71 | 169 |
| Right post. Insula | 48 | -30 | 15 | 3.52 | 193 |
| Left occipital lobe | -15 | -70 | 36 | 3.33 | 94 |
| Area 17 | 3 | -102 | -9 | 7.76 | 68 |
| Right cerebellum | 24 | -43 | -50 | 3.63 | 469 |

p_unc_<0.001, *p_FWE_<0.05 Threshold >30Vx; MNI-Montreal Neurological Institute coordinates, T:effectstrength, k_E_:cluster-size; rightsided = ipsilateral, leftsided = contralateral to headache.

**Tab. S3: Detail information on inter(sub)group analysis:**

|  | **MNI-Coordinates** | | | **T** | **k_E_** |
| --- | --- | --- | --- | --- | --- |
|  | **X** | **Y** | **Z** |  |  |
| **o.b. > i.b** | | | | | |
| Right ventral hippocampus | 39 | -10 | -20 | 3,74 | 119 |
| Right mid. Temporal gyrus | 54 | -55 | 18 | 3,64 | 81 |
| **i.b. > o.b.** | | | | | |
| Left inferior frontal (DLPF) gyrus | -54 | 27 | 15 | 4,39 | 431 |
| Right inferior frontal (DLPF) gyrus | 50 | 18 | 25 | 4,15 | 402 |
| Left anterior insula | -39 | 9 | -5 | 3,62 | 287 |
| Right anterior insula | 39 | 14 | -3 | 3,32 | 40 |
| Left middle frontal gyrus | -44 | 54 | 3 | 3,32 | 68 |
| **o.b. > cCH** | | | | | |
| Left inferiror parietal lobe (S2) | -41 | -51 | 52 | 3,55 | 96 |
| Left dorsal hippocampus | -20 | -33 | -2 | 3,48 | 189 |
| Posterior ACC | -2 | 5 | 24 | 3,48 | 49 |
| Right inferior frontal (orbitofrontal) gyrus | 24 | 14 | -24 | 3,46 | 57 |
| Left inferior frontal (orbitofrontal) gyrus | -23 | 17 | -21 | 3,34 | 75 |
| Left inferior temporal gyrus | -66 | -24 | -17 | 3,40 | 325 |
| Right middle temp gyrus | 62 | -54 | 12 | 3,23 | 32 |
| **cCH > o.b** | | | | | |
| none | | | | | |
| **i.b. > cCH** | | | | | |
| Left cerebellum | -44 | -73 | -35 | 4,0 | 907 |
| Right cerebellum | 41 | -76 | -35 | 3,44 | 106 |
| Left inferior parietal lobe (S2) | -26 | -48 | 45 | 3,46 | 42 |
| Left anterior insula | 38 | 17 | -2 | 3,37 | 107 |
| **cCH > i.b.** | | | | | |
| none | | | | | |

p_unc_<0.001, *p_FWE_<0.05 Threshold >30Vx; MNI-Montreal Neurological Institute coordinates, T:effectstrength, k_E_:Cluster-size; rightsided = ipsilateral, leftsided = contralateral to headache.
